# Distal femoral morphology as a risk factor for osteoarthritis

**DOI:** 10.1002/ar.70012

**Published:** 2025-07-22

**Authors:** Haley Horbaly

**Affiliations:** ^1^ Department of Health and Human Performance Congdon School of Health Sciences, High Point University High Point North Carolina USA; ^2^ Department of Physician Assistant Studies Congdon School of Health Sciences, High Point University High Point North Carolina USA

**Keywords:** articular morphology, knee joint shape, morphological variation, total knee arthroplasty (TKA)

## Abstract

Osteoarthritis (OA) is a leading cause of disability affecting millions of adults in the United States, commonly resulting in the need for total knee arthroplasty (TKA) to restore mobility and quality of life. This study investigates potential differences in baseline distal femoral shape between individuals who received TKA due to OA and a control group representing a healthy population. Using three‐dimensional geometric morphometrics, distal femoral shape was examined in 43 adult skeletons from the University of Tennessee Donated Skeletal Collection. Results suggest that natural femoral shape in TKA‐receiving individuals may differ from that of the control group, with some individuals in the TKA sample occupying more extreme regions of the femoral shape space. In particular, the landmarks of the medial condyle appear anteriorly shifted in the TKA sample, identifying this region as a candidate location for future exploration into group differences. While future longitudinal studies are required to determine direct causal links between morphology and OA as a health outcome, existing clinical literature has identified that even minor mismatch in conarticular shape can alter the biomechanical environment of the joint. These results are a first step in identifying outliers for femoral morphology and potential regions of femoral anatomy that may predispose individuals to OA, highlighting the importance of evaluating morphological variations as potential risk factors. This study further contributes to our understanding of the boundaries of articular morphospace and its implications for arthropathy, underscoring the need for further research to establish direct links between baseline articular shape and OA onset.

## 1 INTRODUCTION

Osteoarthritis (OA) is the most common arthritis variant and is characterized by the breakdown of joint articular cartilages and associated changes to the underlying bone (Lawrence et al., 2008). With no cure for OA, many patients must seek surgical remedies to regain mobility and quality of life. Among patients with OA who seek joint replacement surgery, over half require total knee arthroplasty (TKA; The American Joint Replacement Registry, 2020). Given its location in the limb, knee OA is known to have proliferative effects, often impacting the ipsilateral hip joint (Nam et al., 2016). Diagnosis of OA is commonly based on semi‐quantitative radiographic methods that include a five‐point scale assessment of cartilage loss and subsequent joint space narrowing, osteophyte presence, and evaluation of subchondral bone, among other attributes (Kellgren & Lawrence, 1957). While this approach is a field‐wide standard for tracking the onset and progression of OA, it is not designed to proactively identify baseline features of the knee that may potentially predispose an individual to an OA diagnosis and/or an eventual need for TKA. With OA prevalence only expected to increase in future decades due to increasing lifespans of aging generations and a desire for quality‐of‐life improvements (Cram et al., 2012; Hootman et al., 2016; Kurtz et al., 2009), diagnosing and preventing joint degeneration is a top priority for the healthcare industry.

Onset and progression of knee OA is often associated with biomechanical factors such as joint overuse, conarticular malalignment, altered gait, and uneven wear of articular cartilage (Allen et al., 2022). While an individual's natural knee morphology is not currently part of the OA risk‐factor assessment protocol, even a slight morphological mismatch between conarticulars is known to contribute negatively to the quality of the joint's biomechanical environment and load distribution (Brandt et al., 1991; Brouwer et al., 2007; Kippel & Dieppe, 1994; Matsumoto et al., 2015; Sharma, 2007; Sharma et al., 2010; Stone & Yu, 1997; von Eisenhart‐Rothe et al., 1997), suggesting that variation in articular shape is an important factor in the predisposition of individuals to OA. Of course, OA‐induced degeneration of the joint can result in changes to articular morphology over the lifespan, but whether the baseline pre‐pathological morphology acts as a contributing factor for that degeneration has been incompletely explored. Research on idiopathic joint shape has predominantly revolved around the hip joint, where grossly obvious variations in acetabulofemoral structure have been linked to OA‐promoting conditions such as Perthes' disease, developmental dysplasia, and femoroacetabular impingement (Hatzikotoulas et al., 2018; Lehmann et al., 2012; Wiberg, 1939; Wilkinson & Zeggini, 2021). The knee, however, is one of the most morphologically complex joints in the body, and yet baseline knee shape has received considerably less attention as a possible OA risk factor.

This study uses a modern human skeletal sample to investigate (1) whether natural femoral morphology can distinguish individuals with a history of OA (represented here by individuals with an OA‐induced unilateral TKA) from healthy individuals, and if so, (2) what are the specific morphological features driving these differences. This exploration will help determine if certain variations in distal femoral shape can serve as indicators for OA risk, providing valuable insights for early detection and preventive strategies.

### 1.1 Variation in articular morphology

There are many factors that contribute to skeletal variation in a population, some of which are intrinsic (e.g., caused by variation in developmental processes, timing, or genomic expression) and others extrinsically induced (e.g., adaptation to mechanical loading over time). Intrinsic mechanisms that contribute to articular morphogenesis and variation are incompletely understood (Chijimatsu & Saito, 2019; Kan et al., 2013; Pacifici et al., 2005; Pacifici et al., 2006; Zhang et al., 2020), though there is evidence to suggest that variance‐generating processes deploy in a way that produces high consistency between the articular surfaces of antimeric limb segments (Horbaly & Hubbe, 2024). With regard to extrinsic factors, a body of literature indicates that mechanical input is essential for proper joint development in utero and throughout post‐natal growth (Carter & Wong, 1988; Chijimatsu & Saito, 2019; Frost, 1979; Hamrick, 1999; Plochocki et al., 2009), causing considerable shape changes to articular surfaces throughout ontogeny. However, external articular morphology may not significantly change in response to mechanical loading over an adult's lifetime (Auerbach & Ruff, 2006; Lazenby et al., 2008; Ruff, 1994, 2002), suggesting much of joint shape variation is finalized during growth and development.

Prior work has established a baseline understanding of adult patterns of morphological variation in the articular surfaces of contemporary humans, including the existence of (1) higher morphological variance in concave articular surfaces; (2) higher variance in joints with greater ranges of motion; and (3) higher conarticular covariance in joints with complex morphologies (Horbaly, 2023; Horbaly et al., 2023). Therefore, as a convex articular surface that is part of a morphologically complex joint with lower range of motion relative to the shoulder and hip, for example, the distal femur is found to be relatively morphologically constrained across a population compared to other limb bone articular surfaces (Horbaly et al., 2023). Because these patterns were established for a non‐pathological sample of human joint surfaces, the narrow range of distal femoral variation presumably represents a constrained distribution of shapes that can be healthily tolerated for the joint. As such, slight differences in this morphologically constrained surface could quickly place an individual at the extreme ends of this narrow variance distribution, potentially resulting in an idiopathic mismatch with its conarticular. Accessory fibrocartilaginous structures such as menisci cannot be ignored when discussing articular interaction, as these structures serve to partially enhance congruence between conarticulars (Almajed et al., 2022; McDermott et al., 2008; Walker & Erkman, 1975), among other roles such as load diffusion (Ahmed et al., 1983; Fukubayashi & Kurosawa, 1980; Gee & Posner, 2021) and movement stabilization (Almajed et al., 2022; Bendjaballah et al., 1998; Levy et al., 1982; Levy et al., 1989; Shoemaker & Markolf, 1986). While any bony mismatch may theoretically be accommodated by the presence of accessory fibrocartilaginous structures such as the menisci, conarticular shape covariances do not clearly support this hypothesis (Horbaly, 2023). Evidence that soft tissue padding does not wholly compensate for bony mismatch is further supported by the clinical literature that indicates even subtle mismatches in bony shape can result in joint degeneration (Brandt et al., 1991; Brouwer et al., 2007; Kippel & Dieppe, 1994; Matsumoto et al., 2015; Sharma, 2007; Sharma et al., 2010; Stone & Yu, 1997; von Eisenhart‐Rothe et al., 1997).

### 1.2 Joint shape as an OA risk factor

Previous work has explored the question of idiopathic joint shape more directly, with an emphasis on the hip joint (Gregory et al., 2007; Lynch et al., 2009; Wilkinson & Zeggini, 2021). Early studies identified that underlying variations on acetabulofemoral shape precede the onset of hip joint degeneration in more than 90% of individuals with hip OA (Harris, 1986; Murray, 1965; Solomon, 1976). More recently, Castaño‐Betancourt et al. (2013) demonstrated that the probative value of hip geometry in assessing OA risk is comparable to often‐cited demographic risk factors. Considerably less attention has been given to knee shape and OA risk, though a growing body of literature has begun to investigate this relationship. Wise et al. (2018) used 2D radiographic analysis and found that while females in their sample had a higher risk of OA incidence as a group than males, certain tibial and femoral attributes (such as relative elevation of the tibial condyles and orientation of femoral articulations relative to the diaphysis) appear to mitigate this risk. This suggests the general shape differences between sex groups in the sample may contribute to differential risk of OA. Other 2D radiographic studies such as Haverkamp et al. (2011) have found that a wider distal femur and more elevated lateral tibial plateau were associated with the presence of radiographic OA. While such studies make progress toward establishing whether knee shape is causal for OA, many of the identified patterns are more related to the *size* of specific knee attributes rather than true shape. Further, low‐resolution 2D imaging methods have been known to distort the true morphology (Blake et al., 1999) making shape inferences from this data difficult. Even fewer studies have applied 3D methodology to idiopathic knee shape. Morales Martinez et al. (2020) used a 3D shape modeling approach using MRI images of the knee, and while their results show promise for the ability of deep learning models to distinguish generalizable representations of bone shape, this method does not identify specific articular shape qualities that may predispose an individual to disease onset.

This paper utilizes 3D landmark data from a modern human skeletal collection to build on the existing literature concerning the idiopathic nature of baseline articular morphology, with a specific focus on the knee. It is hypothesized that (1) the distal femora of individuals who received an OA‐induced TKA will show morphological variations that distinguish them as outliers relative to the control group with no history of OA; and (2) specific and identifiable landmarks associated with discrete features of the distal femur will have greater contributions to group differences. These hypotheses are tested here by analyzing the shape of the unreplaced, non‐pathological antimere of individuals who have received a unilateral TKA, as prior research demonstrates that left and right articular surfaces are highly consistent in their deviations from a morphological mean (Horbaly & Hubbe, 2024). This anticipation of morphological similarity between antimeres raises a critical question: if both knees share a similar morphology, why does OA develop in one and not the other? An important distinction should be made between joint shape as a predisposition versus a direct cause. Numerous factors such as biomechanical loading patterns, injury history, or systemic health conditions likely interact with morphology to influence OA onset. Indeed, many pathologies manifest unilaterally despite symmetry across the body, illustrating that anatomical predispositions are not deterministic. The present study proposes that specific femoral morphologies may be one such risk factor that increases the likelihood of OA but does not guarantee its occurrence. This study does not aim to quantify the exact risk or isolate shape as a singular cause; rather, it explores the relevance of morphology in the broader context of OA etiology.

## 2 MATERIALS AND METHODS

Three‐dimensional geometric morphometrics are used here to explore local shape variation that may differ between individuals in the TKA‐receiving population and those who did not receive arthroplasty. Forty‐three adult skeletons from the University of Tennessee Donated Skeletal Collection were examined. Twelve of these individuals were recipients of unilateral TKA, and had antimeric distal femora that exhibited no macroscopic signs of pathology (e.g., lipping of articular surfaces, eburnation, bone erosion); these unreplaced and otherwise healthy‐looking antimeres are the femora under study for the TKA group. The remaining 31 individuals (the control group) had no knee replacements nor signs of pathology and were selected to reflect comparable sex ratios and age ranges relative to the TKA group and therefore was not a random sample. There were no significant group differences (*p* > 0.05) in stature and weight during life, as indicated by *t* tests (Table S1, Supporting Information). Demographic summaries, stature, and weight information for the sample are provided in Table 1.

**TABLE 1:** Sample demographic summary.

	Min	1st Qu.	Median	Mean	3rd Qu.	Max
Non‐TKA (*n* = 31)						
Age	49	65	71	71.9	81	91
Stature (cm)	147.3	160.0	170.2	169.6	180.3	193.0
Weight (lbs)	105.0	128.5	142.5	156.0	166.5	280
TKA (*n* = 12)						
Age	49	67.2	74	73.2	83.5	91
Stature	157.5	160.0	172.7	171.3	177.8	188.0
Weight	95.0	157.5	190.0	186.2	230.8	240.0

Abbreviation: Qu, Quantile.

**FIGURE 1:**
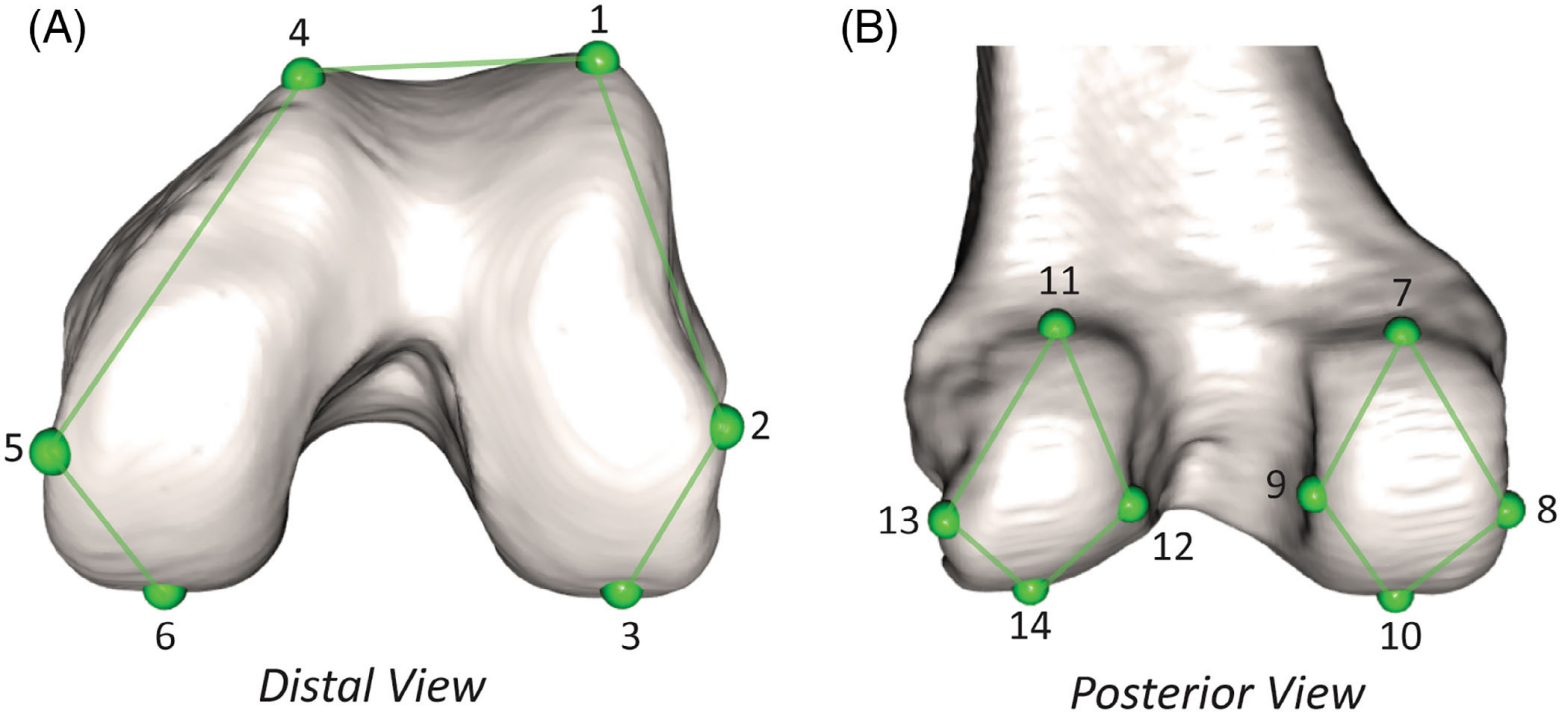
Illustration of distal femur landmarks, demonstrated on the left femur.

For the control group, only left‐side distal femora were used. The TKA group consisted of six left distal femora and six right distal femora. Fourteen 3D landmarks were collected using an Immersion Microscribe GX2 on the distal articular surface of all bony femora. Landmarks were selected to approximate the major features of the femoral articular border, including points of greatest projection and curve inflection. Individual landmark definitions are detailed in Table S2 and illustrated in Figure 1. To test for intraobserver error in landmark placement, all control group landmarks were collected twice in two placement trials conducted a week apart. A paired *t* test for each landmark demonstrates no significant difference (*p* > 0.05 for all pairs) in the placement of 434 landmarks (Table S3). Landmarks collected on the six right femora in the TKA group were reflected across the X‐axis and grouped with the true left femora.

All landmarks were subjected to a full Procrustes transformation (scaling, rotation, and alignment) using the “geomorph” package (Adams et al., 2021; Baken et al., 2021) in R (R Core Team, 2023). A Procrustes ANOVA determined there were no significant differences in shape between sexes (*F* = 1.48, *p* = 0.123, *R* ^2^ = 0.035; see Table S4 for full statistics). This is consistent with prior research (Grelsamer et al., 2005; Horbaly et al., 2023) that indicates no significant differences in morphological variance between male and female distal femora when using Procrustes‐transformed landmarks, thus sexes were pooled for all analyses.

Principal component analysis (PCA) was used to explore the first hypothesis that distal femoral morphology is distinctive among individuals with unilateral TKA. The effects of size on group differences were tested in two ways. First, a linear model was used to test the association between centroid size and principal component (PC) scores to determine if differences along the PC1 axis are due to size‐related variations in shape. Second, PC scores were tested for correlations with living stature and weight data for the sample using Pearson correlations. Direct tests for group differences between the TKA and control samples were conducted using *t* tests to examine whether the means of individual principal components (PC1 and PC2) differ between groups, along with a Procrustes ANOVA to evaluate overall shape differences. While these methods provide insights into global and major‐axis variation, they may not fully capture complex group distinctions. To better assess potential differences across the full set of shape variables, a canonical variates analysis (CVA) was performed using the “candisc” package in R (Friendly & Fox, 2024). Because CVA relies on estimation of within‐group covariance matrices, and the number of shape variables (14 landmarks × 3 dimensions) exceeds the size of each group (TKA *n* = 12; control *n* = 31), it was not feasible to perform CVA directly on Procrustes coordinates. Instead, the PCA previously conducted on Procrustes‐transformed coordinates was used to reduce dimensionality, and the first 21 PCs were retained, which together explained approximately 95% of the total shape variance. These PC scores were used as input to a multivariate linear model with group membership (TKA vs. control) as the independent variable. The candisc() function was then used to extract a canonical variates that maximize between‐group variance relative to within‐group variance.

To evaluate how well shape differences could be used to predict group membership, a linear discriminant analysis (LDA) was performed on the same set of retained PC scores using the lda() function from the “MASS” package in R (Venables & Ripley, 2002). This step allowed for a formal test of classification accuracy, which CVA alone does not provide. Classification performance was evaluated using leave‐one‐out cross‐validation.

To further explore the first hypothesis and better understand whether TKA‐receiving individuals were relative outliers among the sample's total distribution of femoral morphology, a rank‐score analysis was conducted to determine each femur's relative distance from the sample's morphological average. Rank scores were chosen in favor of absolute distances in order to compare the relative positions of the morphologies among the overall distribution, rather than to test whether individuals have significantly different distance values. This is consistent with prior work (Horbaly & Hubbe, 2024) where rank scores are used to demonstrate how “extreme” a morphology is among the sample distribution. Procrustes distances from the centroid were calculated for each femur and were then rank‐ordered, with the lowest rank score (rank = 1) assigned to the femur falling closest to the centroid, and the highest rank score (rank = 43) assigned to the femur falling furthest from the centroid. A Wilcoxon rank‐sum test was performed to determine group differences in rank scores.

A second set of analyses was conducted to test the second hypothesis that group differences in shape can be attributed to specific landmarks, and thus specific anatomical features. Since the aforementioned CVA was conducted on PC scores rather than directly on Procrustes coordinates, the individual loadings of each coordinate onto the discriminant axis (CV1) are not immediately interpretable in anatomical terms. To address this, Pearson correlation coefficients were then computed between CV1 scores and each of the original coordinate variables (*X*, *Y*, *Z* values for each landmark), allowing for identification of landmarks that are most strongly associated with group differences. This process aids in interpreting how specific shape components relate to the classification results.

## 3 RESULTS

**FIGURE 2:**
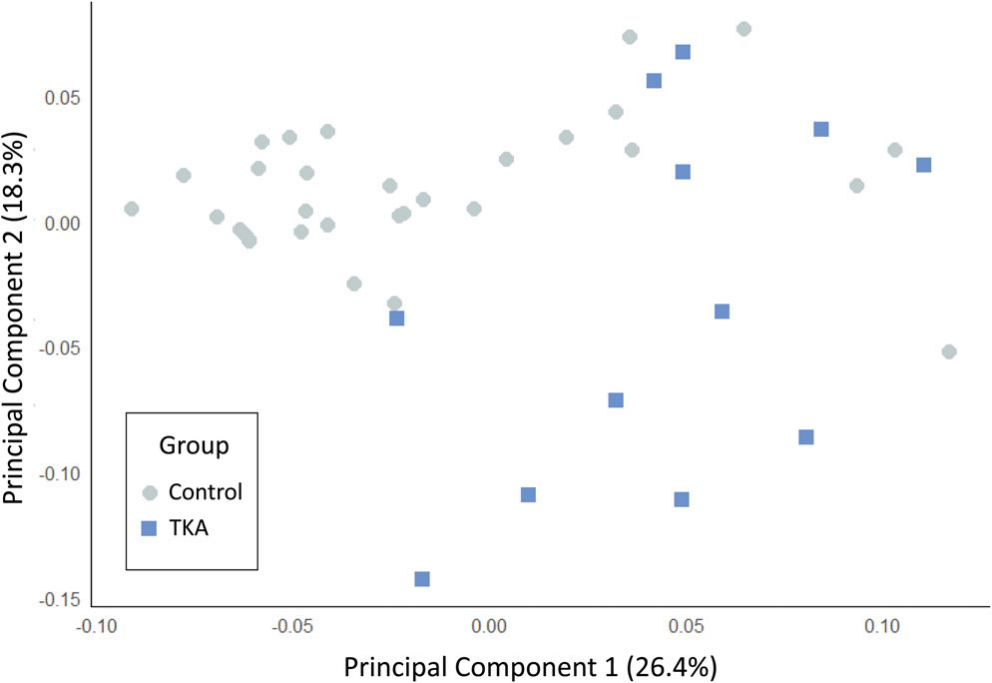
Principal components plot of TKA and control group distal femora. X‐ and Y‐axes represent the first two PCs and their respective amounts of explained variance.

Differences between the TKA and control groups were first explored using PCA. A scree plot determined that the inflection point occurs after the fourth principal component; however, visualizations of PC3 and PC4 (Figure S1) reveal substantial overlap between groups and minimal contribution to the overall explained variance. The first two PCs explain 44.7% of the sample variance, and thus the remaining analysis focuses on PC1 and PC2. There is considerable overlap between distal femora of most TKA and control individuals along the axes of greatest variance, but there is apparent separation between the groups on the diagonal (Figure 2). The effects of size on the PC plot were tested in two ways. First, a linear model was used to evaluate whether centroid size was driving group differences observed along PC1; yet no significant relationship was found between the two variables (*p* = 0.636; Table 2). Second, Pearson correlation tests between body size variables and PC1 and PC2 scores show that neither donor stature nor weight has a significant correlation with PC scores (*p* > 0.05 for all tests; Table 3). Overall, body size and size‐related shape appear to have no relationship with the PC patterns observed here; other demographic factors such as sex and age can also be ruled out as driving the changes observed across PC axes, as the control group was selected to have comparable sex and age structure relative to the TKA group.

**TABLE 2:** Results of linear model evaluating whether centroid size drives group differences along PC1.

Predictor	Estimate	*SE*	*t*‐value	*p*‐value
Intercept	−0.33	0.80	−0.42	0.68
Centroid size	<0.01	<0.01	0.48	0.64

**TABLE 3:** Pearson correlations for association between body size and PC scores.

	*r*	*t*	*p‐*value
PC1 ~ Stature	0.18	1.07	0.29
PC1 ~ Weight	0.33	1.98	0.06
PC2 ~ Stature	0.27	1.67	0.10
PC2 ~ Weight	−0.07	−0.40	0.69

**FIGURE 3:**
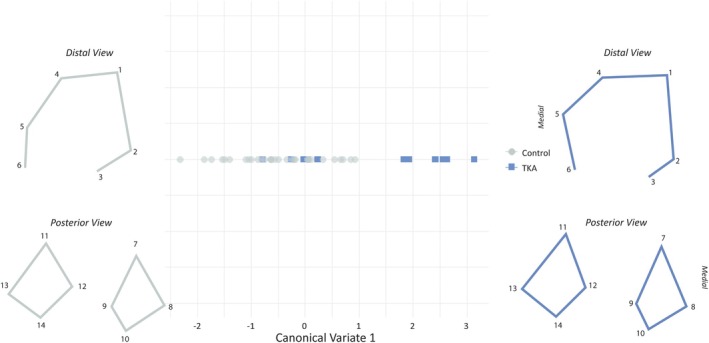
Plot demonstrating group separation along Canonical Variate 1. Wireframes represent shape differences at the extreme ends of CV1.

After initial explorations of the PC plot, groups were directly tested for differences were directly tested for differences in the major shape axes. A series of *t* tests demonstrate that TKA and control groups exhibit no significant differences in PC scores along PC1 nor PC2 (*p* > 0.05 for both tests; Table 4). A Procrustes ANOVA was performed to test for significant differences in shape between the groups. However, no significant group differences were detected (*F* = 0.58, *p* = 0.93; Table 5), a finding that is consistent with the visual overlap on the PC plot. Collectively, the *t* tests and Procrustes ANOVA suggest group differences are either not prominent in the global shape space or may be subtle and not well represented by the primary axes. A canonical variates analysis (CVA) was conducted to further explore potential differences, as this technique is designed to maximize separation between groups along axes of greatest between‐group variation. Because only two groups were included, a single canonical variate (CV1) was extracted. CV1 accounted for 40.6% of the total variance in the PC scores attributable to group differences (*CanRsq* = 0.41, eigenvalue = 0.68). As only one canonical axis can be extracted for a two‐group comparison, CV1 captures 100% of the explainable between‐group variance in this analysis. Class means for the canonical variate were −0.50 and 1.30, indicating separation between groups (Figure 3). The standardized coefficients for the principal components contributing to CV1 are presented in Table S5, with PC7 (0.70) and PC16 (0.39) contributing the most to the discrimination. While these results suggest that group‐level differences in shape may exist, the overlap between individuals along CV1 indicates the differences are relatively subtle and not sufficient for robust group separation.

**TABLE 4:** *t* test results for group differences in PC scores.

	*t*	df	*p*‐value
Group ~ PC1	−0.63	19.50	0.54
Group ~ PC2	−1.44	22.88	0.38

**TABLE 5:** Results of Procrustes ANOVA testing shape differences between TKA and Control groups.

Effect	df	Sum of squares	Mean square	*R* ^2^	*F*	*Z*	*p*‐value
Group	1	0.33	0.33	0.01	0.58	−1.44	0.93
Residuals	41	23.54	0.57	0.99			
Total	42	23.87					

To explore whether individuals could be classified based on shape, a linear discriminant analysis (LDA) model was applied using the same PC scores used in the CVA. Leave‐one‐out cross‐validation (LOOCV) was implemented to evaluate classification accuracy. The model achieved a perfect classification rate by correctly assigning all individuals to the TKA or control groups. However, given the small sample size and moderate group overlap observed in the CVA, this result should be interpreted with caution, as perfect classification under LOOCV may reflect overfitting rather than strong, generalizable group distinctions. Therefore, while the LDA suggests that shape information may contain signals relevant to group membership, these results require validation in a larger dataset.

**FIGURE 4:**
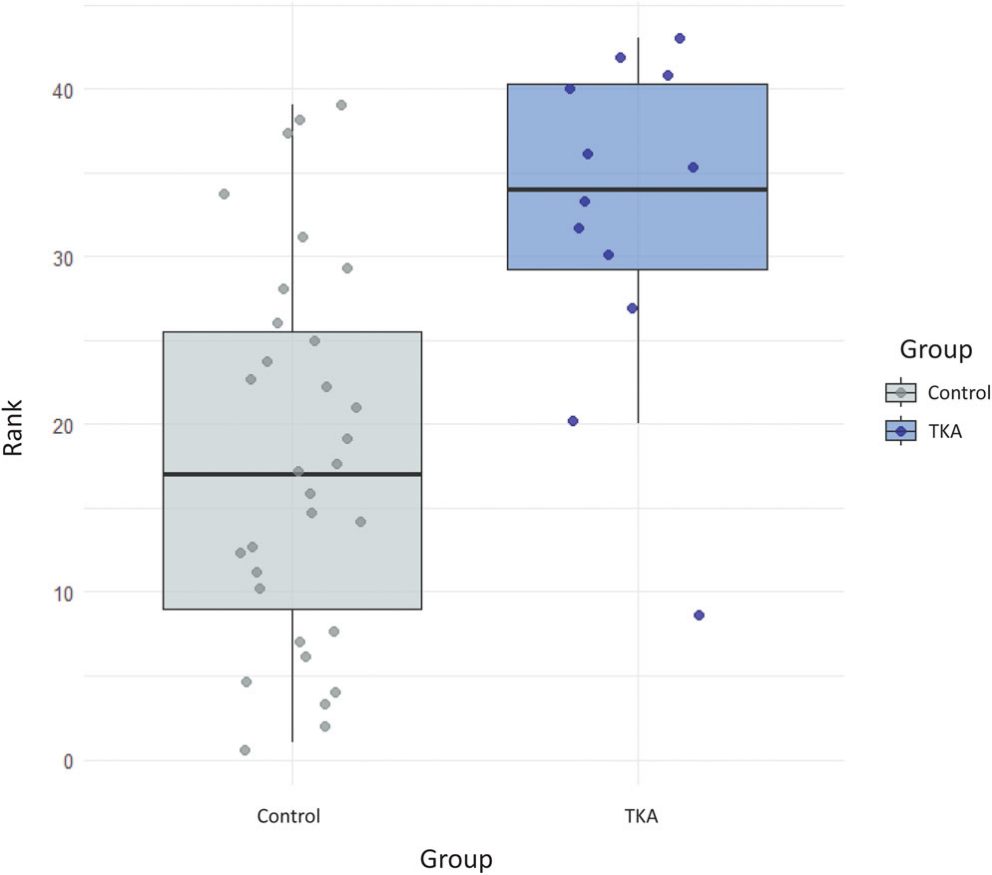
Boxplot of TKA and control group rank scores. Higher rank scores indicate femoral shapes that are further from the morphological average.

A rank‐score analysis was conducted to determine whether the TKA femora were general outliers for shape among the total sample. Each femur in the total sample was assigned a rank numbered 1 (closest) through 43 (farthest) based on Procrustes distance from the mean shape. A Wilcoxon rank‐sum test indicated that rank scores differed significantly between TKA and control groups (*W* = 62, *p* < 0.01) with the TKA group having a higher median rank by 16 positions (Hodges‐Lehmann estimate = 16). This suggests that, on average, femora in the TKA group are more divergent from the morphological mean than those in the control group. However, a boxplot (Figure 4) of the rank scores for each group shows substantial overlap, with some TKA femora ranking close to or even below the control group median. These findings suggest that while TKA individuals tend to deviate more from the average shape, variation within each group is considerable.

The second hypothesis aims to explore whether certain landmarks can be identified as contributing to any patterns observed in the sample. The correlations between the first canonical variate and the landmark coordinates were computed to determine which landmarks most strongly contribute to group differentiation. The correlation values range from −0.68 to 0.53, with many exhibiting significant associations with CV1 (Table 6). Among the anatomical regions analyzed, the femur's medial condyle exhibited the highest proportion of landmarks showing a statistically significant relationship with CV1, suggesting a particularly strong association between this region and the primary axis of group differentiation.

**TABLE 6:** Correlation values between LD1 and landmark coordinates.

Bone feature	Landmark	*X*	*Y*	*Z*
Patellar surface and lateral condyle (distal view)	1	0.17	0.24	0.29
	2	**0.31**	**−0.39**	−0.16
	3	−0.21	**0.45**	**0.40**
Patellar surface and medial condyle (distal view)	4	0.19	−0.22	**0.51**
	5	−0.14	−0.29	**0.47**
	6	**−0.47**	−0.04	0.15
Medial condyle (posterior view)	7	**0.29**	−0.05	0.03
	8	0.08	**0.52**	**−0.33**
	9	0.02	**0.30**	**−0.68**
	10	**−0.32**	**−0.57**	−0.05
Lateral condyle (posterior view)	11	0.00	**0.48**	0.14
	12	0.06	**0.53**	0.06
	13	**0.31**	0.26	**−0.38**
	14	−0.17	−0.13	0.00

*Note*: Significant correlations are bolded. Encased cells represent an absolute value falling within the top 10%.

## 4 DISCUSSION

Prior studies have established associations between both 2D and 3D joint shape data and osteoarthritis, yet specific articular shape qualities that drive these relationships remain elusive. The present study takes a geometric morphometric approach to better illustrate (1) whether the distal femora of unilateral TKA‐receiving individuals are morphologically distinguishable from those of a healthy control sample, and if so, (2) what aspects of distal femoral morphology may be driving group differences. The individuals in the two groups were sex‐ and age‐matched to reduce the effect that these demographic factors may have on resulting patterns; statistical testing confirmed body size variables stature and weight were also not significantly different between the groups (Table S1), though the TKA individuals were, on average, more massive than the control group (Table 1). Altered gait in the wake of unilateral knee replacement has been shown to have both protective (Parisi et al., 2018; Wang et al., 2024) and adverse (Aljehani et al., 2019) impacts on the contralateral knee joint; however, given that the sample selection process confirmed that the antimeric knees of the TKA individuals did not exhibit obvious macroscopic changes associated with osteoarthritis, the patterns observed here are likely not the result of pathological remodeling of bone. Further, because existing literature suggests that articular surfaces are relatively constrained with regard to their propensity for functional adaptation throughout life (Auerbach & Ruff, 2006; Lazenby et al., 2008; Ruff, 1994, 2002), any shape differences between groups are more likely a product of morphogenic processes during growth and development.

The results of the present study provide preliminary evidence for the first hypothesis by demonstrating that subtle group differences can be observed between the TKA and control samples. These differences are modest and nuanced, and do not reach statistical significance in the Procrustes ANOVA, which evaluates overall shape disparity. However, subsequent analyses, including CVA and rank‐based assessments of deviation from the morphological mean, indicate significant separation between groups, and that some individuals in the TKA group exhibit femoral morphologies that deviate more strongly from the overall sample average than do most controls. Given that a modern sample of human femora can be characterized by generally restricted morphological variation (Horbaly et al., 2023), the presence of shape outliers in the healthy knees of the TKA group raises the possibility that innate articular morphology may play a role in OA susceptibility.

Most of the morphological differences between the TKA and control groups occur in the landmarks associated with the femur's medial condyle. A wireframe visualization of the morphological differences across CV1 (Figure 3) shows the most conspicuous morphological differences to be observable from the distal, rather than posterior, view. Specifically, a more anteriorized maximum curvature of the medial condyle, represented by landmark 5, is observed on the TKA end of CV1 relative to the controls. Changes to the position of the medial condyle landmarks also have consequences for the architecture of the patellar surface, and the wireframes demonstrate a slight “leveling out” of this anterior femoral feature. In humans, the lateral patellar surface typically projects further anteriorly than the medial surface, an orientation that maintains proper patellar tracking and prevents a shallowing of the patellar groove known as trochlear dysplasia (Bollier & Fulkerson, 2011; Pfirrmann et al., 2000). The anteriorization of medial condyle landmarks in the TKA group effectively levels the medial and lateral aspects of the patellar surface and reduces groove depth, a morphology commonly observed in patients with trochlear dysplasia and which often results in patellar instability or dislocation (Pennock et al., 2020; Steensen et al., 2015). In addition to reducing stability of the patellofemoral interaction, trochlear dysplasia morphologies have been linked to higher rates of knee joint degeneration and osteoarthritis (De Leissègues et al., 2021; Jungmann et al., 2013).

The anteriorization of medial condylar landmarks and the mild leveling of the patellar surface projections observed in the present study may represent a possible pathway to a pathology‐inducing relationship between the distal femur and patella, and future studies should work toward more thoroughly evaluating such shape features as being part of the causal mechanism for OA onset. In addition to the aforementioned morphological features of trochlear dysplasia, existing clinical research does indeed indicate that even minor differences in the baseline shape of the distal femur are capable of inducing gait patterns or biomechanical load distributions that may promote the onset of degenerative processes like OA (Andriacchi, 1994; Brouwer et al., 2007; Holder et al., 2023; Miyazaki et al., 2002; Morrison, 1970). For example, a typical patella sits centered on the distal femur's trochlear groove, but malalignment of the patella relative to the groove can impart abnormal mechanical strain on the joint and is thought to increase the likelihood of the development of OA (Fujikawa et al., 1983; Harrison et al., 1994; Iwano et al., 1990; Kalichman et al., 2007). Validating and directly linking the architectural differences observed in the present study to the health outcome of osteoarthritis would require longitudinal clinical analysis.

The benefit of understanding differences in the shape of the distal femur between a TKA‐receiving population relative to a control population is two‐fold. First, it provides context to previously established ranges of morphological variation in articular surfaces (Horbaly et al., 2023). Individuals who received an osteoarthritis‐induced total knee arthroplasty appear here as outliers in the morphospace of distal femoral shape. Detecting such deviations from the norm suggests that when femoral shapes approach the edges of the variance distribution, there may be pathological consequences, such as the onset of osteoarthritis. Broadly, this implies that individuals whose articular morphologies fall at the extremes of these distributions may exceed the tolerance of a healthy joint configuration in modern humans, making them more susceptible to arthropathies. Second, recognizing specific articular features that can cause a morphological outlier is crucial for early detection and prevention of osteoarthritis. The results presented here provide valuable insights into localized features that can be observed in a clinical setting, and which may implicate femoral shape as an OA risk factor. The notable anteriorization of medial condylar landmarks may impact the typical relationship between the distal femur and patella.

While this study represents an exploratory step toward understanding the relationship between articular surface shape and susceptibility to joint degeneration, several limitations should be acknowledged. Most notably, the small sample size, particularly in the TKA group, limits statistical power and the broad applicability of the findings. Statistical precautions were taken to work around the small sample size (e.g., conducting CVA on dimension‐reduced PC scores as opposed to directly using the high number of Procrustes coordinates), but the nuanced patterns observed here must be validated with a larger sample. Additionally, this study sample used here is cross‐sectional and aimed at isolating shape features that precede osteoarthritis. Future clinical research, ideally in the form of longitudinal studies, will be necessary to make direct connections between pre‐existing knee morphology and onset of OA.

## 5 CONCLUSION

This study provides preliminary evidence that individuals who have undergone TKA due to osteoarthritis may exhibit subtle morphological differences in their distal femora compared to a control population. While group differences were nuanced, the observed anteriorization and medial rotation of the TKA group's medial condyles suggest that some individuals may fall outside the typical morphospace for distal femoral shape. These deviations could reflect underlying morphological variation that may contribute to OA susceptibility, emphasizing the need for early identification of such morphological outliers. Identifying specific shape features that differ between TKA‐receiving and control individuals is an approach that could eventually inform improved early detection. However, further clinical research is essential to establish definitive links between pre‐existing knee morphology and whether such shape variation has probative value for predicting the onset of OA.

## AUTHOR CONTRIBUTIONS

**Haley Horbaly:** Conceptualization; investigation; writing – original draft; methodology; validation; visualization; writing – review and editing; formal analysis; data curation.

## Supporting information


**Figure S1.** Principal components plot of TKA and control group distal femora. X‐ and Y‐axes represent the third and fourth PCs and their respective amounts of explained variance.

**Data S1.** Supporting Information tables.

## References

[ar70012-bib-0001] Adams, D. C. , Collyer, M. L. , Kaliontzopoulou, A. , & Baken, E. K. (2021). Geomorph: Software for geometric morphometric analyses (Version R package version 4.0.2). Retrieved from https://cran.r-project/org/package=geomorph

[ar70012-bib-0002] Ahmed, A. , Burke, D. , & Yu, A. (1983). In‐vitro measurement of static pressure distribution in synovial joints—Part II: Retropatellar surface. Journal of Biomechanical Engineering, 105, 226–236. 6632824 10.1115/1.3138410

[ar70012-bib-0003] Aljehani, M. , Madara, K. , Snyder‐Mackler, L. , Christiansen, C. , & Zeni, J. A., Jr. (2019). The contralateral knee may not be a valid control for biomechanical outcomes after unilateral total knee arthroplasty. Gait & Posture, 70, 179–184. 30878729 10.1016/j.gaitpost.2019.01.030 PMC8963525

[ar70012-bib-0004] Allen, K. , Thoma, L. , & Golightly, Y. (2022). Epidemiology of osteoarthritis. Osteoarthritis and Cartilage, 30(2), 184–195. 34534661 10.1016/j.joca.2021.04.020 PMC10735233

[ar70012-bib-0005] Almajed, Y. A. , Hall, A. C. , Gillingwater, T. H. , & Alashkham, A. (2022). Anatomical, functional and biomechanical review of the glenoid labrum. Journal of Anatomy, 240(4), 761–771. 34725812 10.1111/joa.13582 PMC8930820

[ar70012-bib-0006] Andriacchi, T. P. (1994). Dynamics of knee malalignment. Orthopedic Clinics of North America, 25(3), 395–403. 8028883

[ar70012-bib-0007] Auerbach, B. M. , & Ruff, C. (2006). Limb bone bilateral asymmetry: Variability and commonality among modern humans. Journal of Human Evolution, 50(2), 203–218. 10.1016/j.jhevol.2005.09.004 16310833

[ar70012-bib-0008] Baken, E. K. , Collyer, M. L. , Kaliontzopoulou, A. , & Adams, D. C. (2021). geomorph v4. 0 and gmShiny: Enhanced analytics and a new graphical interface for a comprehensive morphometric experience. Methods in Ecology and Evolution, 12(12), 2355–2363.

[ar70012-bib-0009] Bendjaballah, M. , Shirazi‐Adl, A. , & Zukor, D. (1998). Biomechanical response of the passive human knee joint under anterior–posterior forces. Clinical Biomechanics, 13(8), 625–633. 11415842 10.1016/s0268-0033(98)00035-7

[ar70012-bib-0010] Blake, G. , Wahner, H. , & Fogelman, I. (1999). Technical principals of X‐ray absorptiometry. In The evaluation of osteoporosis: Dual energy X‐ray absorptiometry and ultrasound in clinical practice (pp. 45–71). Martin Dunitz Ltd.

[ar70012-bib-0011] Bollier, M. , & Fulkerson, J. P. (2011). The role of trochlear dysplasia in patellofemoral instability. American Academy of Orthopaedic Surgeon, 19(1), 8–16. 10.5435/00124635-201101000-00002 21205763

[ar70012-bib-0012] Brandt, K. D. , Myers, S. L. , Burr, D. , & Albrecht, M. (1991). Osteoarthritic changes in canine articular cartilage, subchondral bone, and synovium fifty‐four months after transection of the anterior cruciate ligament. Arthritis & Rheumatism, 34(12), 1560–1570. 1747141 10.1002/art.1780341214

[ar70012-bib-0013] Brouwer, G. , Tol, A. V. , Bergink, A. , Belo, J. , Bernsen, R. , Reijman, M. , Pols, H. A. P. , & Bierma‐Zeinstra, S. (2007). Association between valgus and varus alignment and the development and progression of radiographic osteoarthritis of the knee. Arthritis & Rheumatism, 56(4), 1204–1211. 17393449 10.1002/art.22515

[ar70012-bib-0014] Carter, D. R. , & Wong, M. (1988). The role of mechanical loading histories in the development of diarthrodial joints. Journal of Orthopaedic Research, 6(6), 804–816. 3171761 10.1002/jor.1100060604

[ar70012-bib-0015] Castaño‐Betancourt, M. C. , Van Meurs, J. , Bierma‐Zeinstra, S. , Rivadeneira, F. , Hofman, A. , Weinans, H. , Uitterlinden, A. G. , & Waarsing, J. H. (2013). The contribution of hip geometry to the prediction of hip osteoarthritis. Osteoarthritis and Cartilage, 21(10), 1530–1536. 23811490 10.1016/j.joca.2013.06.012

[ar70012-bib-0016] Chijimatsu, R. , & Saito, T. (2019). Mechanisms of synovial joint and articular cartilage development. Cellular and Molecular Life Sciences, 76(20), 3939–3952. 10.1007/s00018-019-03191-5 31201464 PMC11105481

[ar70012-bib-0017] Cram, P. , Lu, X. , Kates, S. L. , Singh, J. A. , Li, Y. , & Wolf, B. R. (2012). Total knee arthroplasty volume, utilization, and outcomes among Medicare beneficiaries, 1991–2010. Jama, 308(12), 1227–1236. 23011713 10.1001/2012.jama.11153 PMC4169369

[ar70012-bib-0018] De Leissègues, T. , Gunst, S. , Batailler, C. , Kolhe, G. , Lustig, S. , & Servien, E. (2021). Prevalence of trochlear dysplasia in symptomatic isolated lateral patellofemoral osteoarthritis: Transverse study of 101 cases. Orthopaedics & Traumatology: Surgery & Research, 107(7), 102895. 10.1016/j.otsr.2021.102895 33753265

[ar70012-bib-0079] Friendly, M. , & Fox, J. (2024). Candisc: Visualizing Generalized Canonical Discriminant and Canonical Correlation Analysis (R package version 0.9.0). Retrieved from https://CRAN.R-project.org/package=candisc

[ar70012-bib-0019] Frost, H. (1979). A chondral modeling theory. Calcified Tissue International, 28, 181–200. 92358 10.1007/BF02441236

[ar70012-bib-0020] Fujikawa, K. , Seedhom, B. , & Wright, V. (1983). Biomechanics of the patello‐femoral joint. Part II: A study of the effect of simulated femoro‐tibial varus deformity on the congruity of the patello‐femoral compartment and movement of the patella. Engineering in Medicine, 12(1), 13–21. 6682056 10.1243/emed_jour_1983_012_005_02

[ar70012-bib-0021] Fukubayashi, T. , & Kurosawa, H. (1980). The contact area and pressure distribution pattern of the knee: A study of normal and osteoarthrotic knee joints. Acta Orthopaedica Scandinavica, 51(1–6), 871–879. 6894212 10.3109/17453678008990887

[ar70012-bib-0022] Gee, S. M. , & Posner, M. (2021). Meniscus anatomy and basic science. Sports Medicine and Arthroscopy Review, 29(3), e18–e23. 34398117 10.1097/JSA.0000000000000327

[ar70012-bib-0023] Gregory, J. S. , Waarsing, J. H. , Day, J. , Pols, H. A. , Reijman, M. , Weinans, H. , & Aspden, R. M. (2007). Early identification of radiographic osteoarthritis of the hip using an active shape model to quantify changes in bone morphometric features: Can hip shape tell us anything about the progression of osteoarthritis? Arthritis & Rheumatism, 56(11), 3634–3643. 10.1002/art.22982 17968890

[ar70012-bib-0024] Grelsamer, R. , Dubey, A. , & Weinstein, C. (2005). Men and women have similar Q angles: A clinical and trigonometric evaluation. Journal of Bone and Joint Surgery, 87‐B, 1498–1501. 10.1302/0301-620X.87B11.16485 16260666

[ar70012-bib-0025] Hamrick, M. (1999). A chondral modeling theory revisited. Journal of Theoretical Biology, 201, 201–208. 10600363 10.1006/jtbi.1999.1025

[ar70012-bib-0026] Harris, W. H. (1986). Etiology of osteoarthritis of the hip. Clinical Orthopaedics and Related Research, 213, 20–33. 3780093

[ar70012-bib-0027] Harrison, M. M. , Cooke, T. D. V. , Fisher, B. S. , & Griffin, M. P. (1994). Patterns of knee arthrosis and patellar subluxation. Clinical Orthopaedics and Related Research, 309, 56–63. 7994977

[ar70012-bib-0028] Hatzikotoulas, K. , Roposch, A. , Shah, K. M. , Clark, M. J. , Bratherton, S. , Limbani, V. , Steinberg, J. , Zengini, E. , Warsame, K. , Ratnayake, M. , Tselepi, M. , Schwartzentruber, J. , Loughlin, J. , Eastwood, D. M. , Zeggini, E. , & Wilkinson, J. M. (2018). Genome‐wide association study of developmental dysplasia of the hip identifies an association with GDF5. Communications Biology, 1(1), 56. 30273415 10.1038/s42003-018-0052-4 PMC6123669

[ar70012-bib-0029] Haverkamp, D. J. , Schiphof, D. , Bierma‐Zeinstra, S. M. , Weinans, H. , & Waarsing, J. H. (2011). Variation in joint shape of osteoarthritic knees. Arthritis & Rheumatism, 63(11), 3401–3407. 21811994 10.1002/art.30575

[ar70012-bib-0030] Holder, J. , van Drongelen, S. , Uhlrich, S. D. , Herrmann, E. , Meurer, A. , & Stief, F. (2023). Peak knee joint moments accurately predict medial and lateral knee contact forces in patients with valgus malalignment. Scientific Reports, 13(1), 2870. 36806297 10.1038/s41598-023-30058-4 PMC9938879

[ar70012-bib-0031] Hootman, J. M. , Helmick, C. G. , Barbour, K. E. , Theis, K. A. , & Boring, M. A. (2016). Updated projected prevalence of self‐reported doctor‐diagnosed arthritis and arthritis‐attributable activity limitation among US adults, 2015–2040. Arthritis and Rheumatology, 68(7), 1582–1587. 27015600 10.1002/art.39692 PMC6059375

[ar70012-bib-0032] Horbaly, H. (2023). Covariance in human limb joint articular morphology. American Journal of Biological Anthropology, 182(3), 401–411. 10.1002/ajpa.24826 37702982

[ar70012-bib-0077] Horbaly, H. , & Hubbe, M. (2024). Systemic versus local patterns of limb joint articular morphology inferred from relative distances from morphological centroid. Anatomical Record, 307(11), 3519–3528. 10.1002/ar.25506 38817037

[ar70012-bib-0033] Horbaly, H. , Hubbe, M. , Sylvester, A. D. , Steadman, D. W. , & Auerbach, B. M. (2023). Variation in human limb joint articular morphology. American Journal of Biological Anthropology, 182(3), 388–400. 10.1002/ajpa.24829 37702986

[ar70012-bib-0034] Iwano, T. , Kurosawa, H. , Tokuyama, H. , & Hoshikawa, Y. (1990). Roentgenographic and clinical findings of patellofemoral osteoarthrosis: With special reference to its relationship to femorotibial osteoarthrosis and etiologic factors. Clinical Orthopaedics and Related Research, 252, 190–197. 2302884

[ar70012-bib-0035] Jungmann, P. M. , Tham, S.‐C. , Liebl, H. , Nevitt, M. C. , McCulloch, C. E. , Lynch, J. , & Link, T. M. (2013). Association of trochlear dysplasia with degenerative abnormalities in the knee: Data from the Osteoarthritis Initiative. Skeletal Radiology, 42, 1383–1392. 23801099 10.1007/s00256-013-1664-x PMC3757255

[ar70012-bib-0036] Kalichman, L. , Zhang, Y. , Niu, J. , Goggins, J. , Gale, D. , Felson, D. T. , & Hunter, D. (2007). The association between patellar alignment and patellofemoral joint osteoarthritis features—An MRI study. Rheumatology, 46(8), 1303–1308. 10.1093/rheumatology/kem095 17525117

[ar70012-bib-0037] Kan, A. , Toshiyuki, I. , Fukai, A. , Nakagawa, T. , Nakamura, K. , Chung, U. , Kawaguchi, H. , & Tabin, C. J. (2013). Sox11 contributes to the regulation of GDF5 in joint maintenance. Developmental Biology, 13(4), 1–12. 23356643 10.1186/1471-213X-13-4 PMC3760452

[ar70012-bib-0038] Kellgren, J. H. , & Lawrence, J. (1957). Radiological assessment of osteo‐arthrosis. Annals of the Rheumatic Diseases, 16(4), 494. 13498604 10.1136/ard.16.4.494 PMC1006995

[ar70012-bib-0039] Kippel, J. , & Dieppe, P. (1994). Rheumatology. Mosby‐Year Book.

[ar70012-bib-0040] Kurtz, S. M. , Lau, E. , Ong, K. , Zhao, K. , Kelly, M. , & Bozic, K. J. (2009). Future young patient demand for primary and revision joint replacement: National projections from 2010 to 2030. Clinical Orthopaedics and Related Research, 467(10), 2606–2612. 19360453 10.1007/s11999-009-0834-6 PMC2745453

[ar70012-bib-0041] Lawrence, R. C. , Felson, D. T. , Helmick, C. G. , Arnold, L. M. , Choi, H. , Deyo, R. A. , Gabriel, S. , Hirsch, R. , Hochberg, M. C. , Jordan, J. M. , Katz, J. N. , Kremers, H. M. , Wolfe, F. , & Hunder, G. G. (2008). Estimates of the prevalence of arthritis and other rheumatic conditions in the United States: Part II. Arthritis & Rheumatism, 58(1), 26–35. 18163497 10.1002/art.23176 PMC3266664

[ar70012-bib-0042] Lazenby, R. A. , Cooper, D. M. , Angus, S. , & Hallgrímsson, B. (2008). Articular constraint, handedness, and directional asymmetry in the human second metacarpal. Journal of Human Evolution, 54(6), 875–885. 10.1016/j.jhevol.2007.12.001 18207490

[ar70012-bib-0043] Lehmann, T. G. , Engesæter, I. Ø. , Laborie, L. B. , Lie, S. A. , Rosendahl, K. , & Engesæter, L. B. (2012). Total hip arthroplasty in young adults, with focus on Perthes' disease and slipped capital femoral epiphysis: Follow‐up of 540 subjects reported to the Norwegian Arthroplasty Register during 1987–2007. Acta Orthopaedica, 83(2), 159–164. 22112152 10.3109/17453674.2011.641105 PMC3339530

[ar70012-bib-0044] Levy, I. M. , Torzilli, P. , Gould, J. D. , & Warren, R. (1989). The effect of lateral meniscectomy on motion of the knee. Journal of Bone & Joint Surgery, 71(3), 401–406. 2925713

[ar70012-bib-0045] Levy, I. M. , Torzilli, P. , & Warren, R. (1982). The effect of medial meniscectomy on anterior‐posterior motion of the knee. Journal of Bone and Joint Surgery, 64(6), 883–888. 6896333

[ar70012-bib-0046] Lynch, J. , Parimi, N. , Chaganti, R. , Nevitt, M. , Lane, N. E. , & Study of Osteoporotic Fractures Research Group . (2009). The association of proximal femoral shape and incident radiographic hip OA in elderly women. Osteoarthritis and Cartilage, 17(10), 1313–1318. 19427402 10.1016/j.joca.2009.04.011 PMC3678721

[ar70012-bib-0047] Matsumoto, T. , Hashimura, M. , Takayama, K. , Ishida, K. , Kawakami, Y. , Matsuzaki, T. , Nakano, N. , Matsushita, T. , Kuroda, R. , & Kurosaka, M. (2015). A radiographic analysis of alignment of the lower extremities–initiation and progression of varus‐type knee osteoarthritis. Osteoarthritis and Cartilage, 23(2), 217–223. 25481289 10.1016/j.joca.2014.11.015

[ar70012-bib-0048] McDermott, I. D. , Masouros, S. D. , & Amis, A. A. (2008). Biomechanics of the menisci of the knee. Current Orthopaedics, 22(3), 193–201.

[ar70012-bib-0049] Miyazaki, T. , Wada, M. , Kawahara, H. , Sato, M. , Baba, H. , & Shimada, S. (2002). Dynamic load at baseline can predict radiographic disease progression in medial compartment knee osteoarthritis. Annals of the Rheumatic Diseases, 61(7), 617–622. 12079903 10.1136/ard.61.7.617 PMC1754164

[ar70012-bib-0050] Morales Martinez, A. , Caliva, F. , Flament, I. , Liu, F. , Lee, J. , Cao, P. , Shah, R. , Majumdar, S. , & Pedoia, V. (2020). Learning osteoarthritis imaging biomarkers from bone surface spherical encoding. Magnetic Resonance in Medicine, 84(4), 2190–2203. 10.1002/mrm.28251 32243657 PMC7329596

[ar70012-bib-0051] Morrison, J. (1970). The mechanics of the knee joint in relation to normal walking. Journal of Biomechanics, 3(1), 51–61. 5521530 10.1016/0021-9290(70)90050-3

[ar70012-bib-0052] Murray, R. (1965). The aetiology of primary osteoarthritis of the hip. British Journal of Radiology, 38(455), 810–824. 5842578 10.1259/0007-1285-38-455-810

[ar70012-bib-0053] Nam, K. W. , Tsai, T.‐Y. , Dimitriou, D. , Li, G. , & Kwon, Y.‐M. (2016). Ipsilateral varus knee alignment correlates with increased femoral stem anteversion in primary total hip arthroplasty. Hip International, 26(2), 175–179. 26951548 10.5301/hipint.5000325

[ar70012-bib-0054] Pacifici, M. , Koyama, E. , & Iwamoto, M. (2005). Mechanisms of synovial joint and articular cartilage formation: Recent advances, but many lingering mysteries. Birth Defects Research. Part C, Embryo Today, 75(3), 237–248. 10.1002/bdrc.20050 16187328

[ar70012-bib-0055] Pacifici, M. , Koyama, E. , Shibukawa, Y. , Wu, C. , Tamamura, Y. , Enomoto‐Iwamoto, M. , & Iwamoto, M. (2006). Cellular and molecular mechanisms of synovial joint and articular cartilage formation. Annals of the new York Academy of Sciences, 1068, 74–86. 10.1196/annals.1346.010 16831907 PMC2697570

[ar70012-bib-0056] Parisi, T. J. , Levy, D. L. , Dennis, D. A. , Harscher, C. A. , Kim, R. H. , & Jennings, J. M. (2018). Radiographic changes in nonoperative contralateral knee after unilateral total knee arthroplasty. Journal of Arthroplasty, 33(7), S116–S120. 29548619 10.1016/j.arth.2018.02.018

[ar70012-bib-0057] Pennock, A. T. , Chang, A. , Doan, J. , Bomar, J. D. , & Edmonds, E. W. (2020). 3D knee trochlear morphology assessment by magnetic resonance imaging in patients with normal and dysplastic trochleae. Journal of Pediatric Orthopaedics, 40(3), 114–119. 32028472 10.1097/BPO.0000000000001188

[ar70012-bib-0058] Pfirrmann, C. W. , Zanetti, M. , Romero, J. , & Hodler, J. (2000). Femoral trochlear dysplasia: MR findings. Radiology, 216(3), 858–864. 10966723 10.1148/radiology.216.3.r00se38858

[ar70012-bib-0059] Plochocki, J. H. , Ward, C. V. , & Smith, D. E. (2009). Evaluation of the chondral modeling theory using fe‐simulation and numeric shape optimization. Journal of Anatomy, 214(5), 768–777. 10.1111/j.1469-7580.2009.01070.x 19438771 PMC2707100

[ar70012-bib-0060] The American Joint Replacement Registry . (2020). The seventh annual report of the AJRR on hip and knee arthroplasty.

[ar70012-bib-0078] R Core Team . (2023). R: A language and environment for statistical computing. R Foundation for Statistical Computing. https://www.R-project.org/

[ar70012-bib-0061] Ruff, C. (1994). Morphological adaptation to climate in modern and fossil hominids. American Journal of Physical Anthropology, 37, 65–107.

[ar70012-bib-0062] Ruff, C. (2002). Long bone articular and diaphyseal structure in Old World monkeys and apes. I: Locomotor effects. American Journal of Physical Anthropology, 119(4), 305–342. 12448016 10.1002/ajpa.10117

[ar70012-bib-0063] Sharma, L. (2007). The role of varus and valgus alignment in knee osteoarthritis. Arthritis and Rheumatism, 56(4), 1044–1047. 17393411 10.1002/art.22514

[ar70012-bib-0064] Sharma, L. , Song, J. , Dunlop, D. , Felson, D. , Lewis, C. E. , Segal, N. , Torner, J. , Cooke, T. D. , Hietpas, J. , Nevitt, M. , & Lynch, J. (2010). Varus and valgus alignment and incident and progressive knee osteoarthritis. Annals of the Rheumatic Diseases, 69(11), 1940–1945. 20511608 10.1136/ard.2010.129742 PMC2994600

[ar70012-bib-0065] Shoemaker, S. , & Markolf, K. (1986). The role of the meniscus in the anterior‐posterior stability of the loaded anterior cruciate‐deficient knee. Effects of partial versus total excision. Journal of Bone & Joint Surgery, 68(1), 71–79. 3753605

[ar70012-bib-0066] Solomon, L. (1976). Patterns of osteoarthritis of the hip. Journal of Bone & Joint Surgery British Volume, 58(2), 176–183. 932079 10.1302/0301-620X.58B2.932079

[ar70012-bib-0067] Steensen, R. N. , Bentley, J. C. , Trinh, T. Q. , Backes, J. R. , & Wiltfong, R. E. (2015). The prevalence and combined prevalences of anatomic factors associated with recurrent patellar dislocation: A magnetic resonance imaging study. American Journal of Sports Medicine, 43(4), 921–927. 25587185 10.1177/0363546514563904

[ar70012-bib-0068] Stone, J. , & Yu, H. (1997). Computational contact analysis of joint congruency. Biomedical Sciences Instrumentation, 34, 368–373. 9603068

[ar70012-bib-0069] Venables, W. N. , & Ripley, B. D. (2002). Modern applied statistics with S (4th ed.). Springer.

[ar70012-bib-0070] von Eisenhart‐Rothe, R. , Eckstein, F. , Müller‐Gerbl, M. , Landgraf, J. , Rock, C. , & Putz, R. (1997). Direct comparison of contact areas, contact stress and subchondral mineralization in human hip joint specimens. Anatomy and Embryology, 195(3), 279–288. 9084826 10.1007/s004290050047

[ar70012-bib-0071] Walker, P. , & Erkman, M. J. (1975). The role of the menisci in force transmission across the knee. Clinical Orthopaedics and Related Research, 109, 184–192. 10.1097/00003086-197506000-00027 1173360

[ar70012-bib-0072] Wang, H. , Duan, W. , Dang, X. , Chen, Z. , Peng, Y. , Yao, S. , Zhang, W. , & Ma, J. (2024). Kinematic effects of unilateral TKA on the contralateral knee in Chinese patients with advanced osteoarthritis: A prospective gait analysis study. Frontiers in Bioengineering and Biotechnology, 12, 1463049. 39323761 10.3389/fbioe.2024.1463049 PMC11422112

[ar70012-bib-0073] Wiberg, G. (1939). Studies on dysplastic acetabula and congenital subluxation of the hip joint. Acta Chirurgica Scandinavica, 58, 5–135.

[ar70012-bib-0074] Wilkinson, J. M. , & Zeggini, E. (2021). The genetic epidemiology of joint shape and the development of osteoarthritis. Calcified Tissue International, 109(3), 257–276. 32393986 10.1007/s00223-020-00702-6 PMC8403114

[ar70012-bib-0075] Wise, B. L. , Niu, J. , Zhang, Y. , Liu, F. , Pang, J. , Lynch, J. A. , & Lane, N. E. (2018). Bone shape mediates the relationship between sex and incident knee osteoarthritis. BMC Musculoskeletal Disorders, 19(1), 1–9. 30208910 10.1186/s12891-018-2251-z PMC6136224

[ar70012-bib-0076] Zhang, Y. , Annusver, K. , Sunadome, K. , Kameneva, P. , Edwards, S. , Lei, G. , Kasper, M. , Chagin, A. S. , Adameyko, I. , & Xie, M. (2020). Epiphyseal cartilage formation involves differential dynamics of various cellular populations during embryogenesis. Frontiers in Cell and Developmental Biology, 8, 122. 10.3389/fcell.2020.00122 32211405 PMC7066500

