# Supplementary figures and images for: Distal femoral morphology as a risk factor for osteoarthritis
Source: Anat Rec (Hoboken). 2025 Jul 22;309(5):1394–405. doi: 10.1002/ar.70012 (PMC13047956; doi:10.1002/ar.70012)

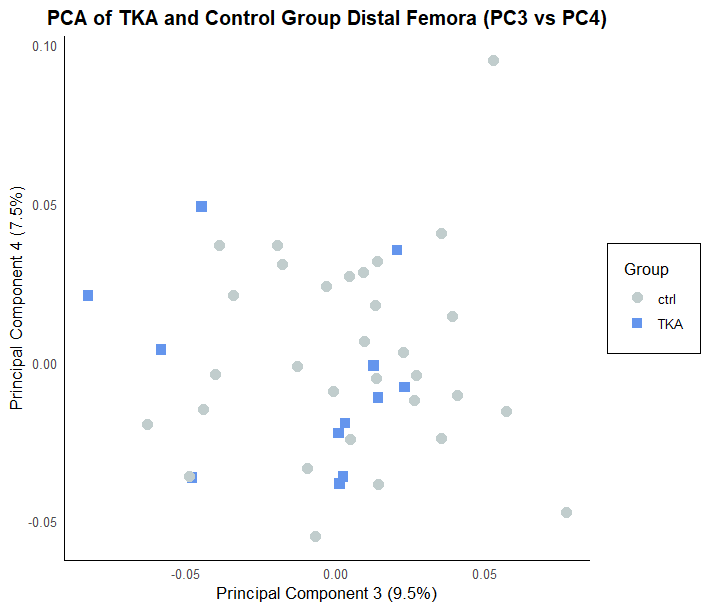

Supplement: Supplementary file 1 — Figure S1. Principal components plot of TKA and control group distal femora. X‐ and Y‐axes represent the third and fourth PCs and their respective amounts of explained variance. [file AR-309-1394-s002.tiff]
