# Supplementary material for: Distal femoral morphology as a risk factor for osteoarthritis
Source: Anat Rec (Hoboken). 2025 Jul 22;309(5):1394–405. doi: 10.1002/ar.70012 (PMC13047956; doi:10.1002/ar.70012)
Supplement: Supplementary file 2 — Data S1. Supporting Information tables. [file AR-309-1394-s001.docx]

Supporting Information

Figure SI.1: Principal components plot of TKA and control group distal femora. X- and Y-axes represent the third and fourth PCs and their respective amounts of explained variance.

Table SI.1: T-tests for group (TKA v. Control) differences in stature and weight

|  | *t* | df | *p*-value |
| --- | --- | --- | --- |
| Group ~ Stature | -0.42 | 20.33 | 0.68 |
| Group ~ Weight | -1.86 | 21.72 | 0.08 |

Table SI.2: Distal femur landmark definitions

| LM # | Location | Description |
| --- | --- | --- |
| 1 | Patellar surface | When viewed distally, the anteriormost projection of the lateral patellar lip |
| 2 | Lateral condyle | When viewed distally, the lateralmost projection of the lateral condyle |
| 3 | Lateral condyle | When viewed distally, the posteriormost projection of the lateral condyle |
| 4 | Patellar surface | When viewed distally, the anteriormost projection of the medial patellar lip |
| 5 | Medial condyle | When viewed distally, the medialmost projection of the medial condyle |
| 6 | Medial condyle | When viewed distally, the posteriormost projection of the medial condyle |
| 7 | Medial condyle | When viewed posteriorly, the superiormost projection of the medial condyle |
| 8 | Medial condyle | When viewed posteriorly, the medialmost projection of the medial condyle |
| 9 | Medial condyle | When viewed posteriorly, the lateralmost projection of the medial condyle |
| 10 | Medial condyle | When viewed posteriorly, the inferiormost projection of the medial condyle |
| 11 | Lateral condyle | When viewed posteriorly, the superiormost projection of the lateral condyle |
| 12 | Lateral condyle | When viewed posteriorly, the medialmost projection of the lateral condyle |
| 13 | Lateral condyle | When viewed posteriorly, the lateralmost projection of the lateral condyle |
| 14 | Lateral condyle | When viewed posteriorly, the inferiormost projection of the lateral condyle |

Table SI.3: Paired t-test results for landmark placement between two trials. T-tests were conducted on Procrustes transformed landmarks.

| Landmark | Mean Difference | t-value | p-value | 95% Confidence Interval |
| --- | --- | --- | --- | --- |
| 1 | 0.0002 | 0.4042 | 0.6889 | (-0.0008, 0.0013) |
| 2 | 0.0002 | 0.2139 | 0.8321 | (-0.0014, 0.0017) |
| 3 | 0.0005 | 0.9193 | 0.3652 | (-0.0006, 0.0017) |
| 4 | -0.0003 | -0.5529 | 0.5845 | (-0.0016, 0.0009) |
| 5 | 0.0015 | 0.9004 | 0.3751 | (-0.0019, 0.0049) |
| 6 | 0.0009 | 1.1753 | 0.2491 | (-0.0006, 0.0024) |
| 7 | 0.0005 | 0.7103 | 0.4830 | (-0.0009, 0.0019) |
| 8 | -0.0022 | -1.9273 | 0.0635 | (-0.0045, 0.0001) |
| 9 | -0.0011 | -0.8759 | 0.3880 | (-0.0037, 0.0015) |
| 10 | 7.61e-05 | 0.0651 | 0.9485 | (-0.0023, 0.0025) |
| 11 | 0.0006 | 1.1504 | 0.2591 | (-0.0005, 0.0018) |
| 12 | -0.0006 | -0.7996 | 0.4302 | (-0.0022, 0.0010) |
| 13 | 0.0014 | 1.8380 | 0.0760 | (-0.0001, 0.0030) |
| 14 | -0.0016 | -1.4280 | 0.1636 | (-0.0040, 0.0007) |

Table SI.4: Results of Procrustes ANOVA testing shape differences between sexes

| Effect | Df | Sum of Squares | Mean Square | R^2^ | F | Z | *p*-value |
| --- | --- | --- | --- | --- | --- | --- | --- |
| Sex | 1 | 0.832 | 0.832 | 0.035 | 1.479 | 1.209 | 0.123 |
| Residuals | 41 | 23.050 | 0.562 | 0.965 |  |  |  |
| Total | 42 | 23.882 |  | 1.000 |  |  |  |

Table SI.5: Standardized Coefficients for Principal Components used in CVA

| PC | Standardized Coefficient |
| --- | --- |
| PC1 | 0.200 |
| PC2 | 0.413 |
| PC3 | 0.076 |
| PC4 | -0.071 |
| PC5 | 0.224 |
| PC6 | 0.011 |
| PC7 | 0.701 |
| PC8 | 0.045 |
| PC9 | 0.088 |
| PC10 | 0.165 |
| PC11 | -0.016 |
| PC12 | 0.116 |
| PC13 | -0.171 |
| PC14 | -0.177 |
| PC15 | 0.307 |
| PC16 | 0.391 |
| PC17 | -0.214 |
| PC18 | -0.400 |
| PC19 | 0.160 |
| PC20 | 0.341 |
| PC21 | 0.323 |
